# Crystal Dehydration in Membrane Protein Crystallography

**DOI:** 10.1007/978-3-319-35072-1_6

**Published:** 2016-04-28

**Authors:** Juan Sanchez-Weatherby, Isabel Moraes

**Affiliations:** 1 Diamond Light Source, Harwell Science and innovation Campus, Didcot, OX11 0DE UK; 2 Membrane Protein Laboratory, Diamond Light Source/Imperial College London, Harwell Campus, Didcot, Oxfordshire UK

**Keywords:** Crystal dehydration, HC1, Free mounting system, *In situ*, Membrane proteins, Relative humidity

## Abstract

Crystal dehydration has been successfully implemented to facilitate the structural solution of a number of soluble and membrane protein structures over the years. This chapter will present the currently available tools to undertake controlled crystal dehydration, focusing on some successful membrane protein cases. Also discussed here will be some practical considerations regarding membrane protein crystals and the relationship between different techniques in order to help researchers to select the most suitable technique for their projects.

## Introduction

The effect of Dehydration in crystal diffraction has been known since the beginnings of protein crystallography (Perutz 1946). It can often be detrimental to the crystal but also be very beneficial, dramatically improving diffraction properties. Partly exacerbated by the initial unavailability of cryo-cooling techniques, and tight control of water content in crystals prior or during data collection, dehydration has always been of paramount importance. In the early days, when most data were collected at room temperature, it was necessary to maintain the stability of samples for hours or even days (Pickford et al. 1993). This meant that researchers were more aware of the benefits and pitfalls of hydration control and accustomed to accounting for the role it played in their observed results. Dehydration has been reported to be beneficial on the diffraction quality of macromolecular crystals (Heras and Martin 2005; Newman 2006; Russo et al. 2012). In most cases the discovery has occurred as a result of casual observations (such cracked capillaries, drops left to dry, badly sealed trays and others) (Esnouf et al. 1998; Abergel 2004).

The changes caused by dehydration are of such importance that several past and present studies have focused solely in the understanding of alterations caused by dehydration and its implications on the functional interpretation of protein and DNA structures. (Biswal and Vijayan 2002; Kaushal et al. 2008; Saraswathi et al. 2002; Nagendra et al. 1998; Sukumar et al. 1999; Kuo et al. 2003; Bowler et al. 2006; Perutz 1946; Amunts et al. 2007; Dobrianov et al. 2001).

As X-ray sources became stronger, cryo-cooling rapidly became the norm (Hope 1988; Mitchell and Garman 1994; Garman 1999; Juers and Matthews 2004; Alcorn and Juers 2010). Once crystal samples are cooled, most of the alterations are prevented, so the need for careful control of humidity is diminished and shifted from the data collection stage to the pre-cooling sample preparation step (Farley et al. 2014; Pflugrath 2015). Crystal handling can have a significant effect on the hydration state of samples and, as it has been highlighted recently, it can have potential consequences in the structural solution (Farley et al. 2014). Therefore, crystallographers have a number of techniques and “tricks” in order to prevent these effects with greater or lesser success. Chemically, the cryo-protection process also causes alterations in osmotic pressure in the crystal samples, similar to a hydration/dehydration process, thus it can change the structure and packing, often leading to changes in diffraction quality (Heras and Martin 2005).

Despite documented benefits of controlled dehydration in macromolecular crystallography, dehydration is often not pursued due to a lack of well-established protocols and equipment. To address this, in the last few years a number of novel techniques have been made available, which focus on trying to make the experiments easier to carry out, reproducible and where possible, with X-ray feedback. These techniques range from simple tools to aid capillary mounting (Basavappa et al. 2003; Kalinin et al. 2005; Mac Sweeney and D’Arcy 2003; Yadav et al. 2005), to bespoke equipment designed to maintain a humid environment around the samples while mounted in the beam path of a synchrotron beamline (Sanchez-Weatherby et al. 2009; Russi et al. 2011; Bowler et al. 2015; Kiefersauer et al. 2000a; Kiefersauer et al. 2000b; Kiefersauer etal. 2002; Kiefersauer et al. 2014).

The move to more complex crystallographic challenges and the availability of new techniques has meant more researchers are trying these experiments. As more experiments are being carried out and successes have been coming through, interest in crystal dehydration has been recently reignited (Moraes et al. 2014; Bowler et al. 2015; Kiefersauer et al. 2014).

This chapter will briefly review the current status of the field and the available tools, it will discuss the basic aspects of a dehydration experiment and give some guidance when attempting membrane protein crystal dehydration.

## The Dehydration Method

As noted in the past (Newman 2006), dehydration can be a bit of a confusing term. Here we consider crystal dehydration as any process able to remove available water molecules from the crystals lattice. Dehydration is achieved by either altering the vapour (Sect. 6.2.1) or the chemical (Sect. 6.2.2) equilibrium of the crystal. These two methods of dehydration are different but also interrelated as the chemical alterations can be used to induce a change in the vapour environment and, the changes in the vapour surrounding the sample can induce alterations the chemistry. The relative amount of water present in a volume of air, Relative Humidity (RH), is directly linked to the temperature and chemical nature of a solution in equilibrium with the air. For this reason, salt solutions are universal calibrating standards for any equipment designed to measure air RH (Greenspan 1977).

When dehydrating crystals, it is advisable to try methods based on both approaches. In some cases, both methods can lead to very similar results but in others (particularly membrane protein crystals with Detergents) the outcome of the process can be dependent on the particular technique used.

Crystallographers working in closed systems, like Crystallisation plates or capillaries, can guide their dehydration experiments based on the well-established number of standard salts. Furthermore, the empirical equilibrium relative humidity when using the humidity control device (HC1) (Bowler et al. 2015; Russi et al. 2011; Sanchez-Weatherby et al. 2009) or the free mounting system (FMS) (Kiefersauer et al. 2002; Kiefersauer et al. 2000a, b) can be tabulated and tools exist that allow the researcher to try to estimate the theoretical alterations caused by chemical modification or the formulation of solutions required to induce a particular change in RH (Bowler et al. 2015; Wheeler et al. 2012).

### Dehydration by Modifying the Vapour Equilibrium

This method is based on altering the water vapour pressure of the air surrounding the samples. This makes the water in the surrounding Crystallisation buffer and crystal solvent channels equilibrate with the surrounding air making it saturate the air and lowering its availability in solution. Methods to carry this type of dehydration range from simple exposure of the samples to ambient dry air, through controlling the air environment using chemicals (Hellert et al. 2014; Kalinin et al. 2005; Kalinin and Thorne 2005), to the use of bespoke equipment designed to expose the samples to controlled dehydration (Bowler et al. 2015; Russi et al. 2011; Sanchez-Weatherby et al. 2009; Kiefersauer et al. 2002; Kiefersauer et al. 2000a, b).

### Dehydration by Modifying the Chemical Equilibrium

Chemical alteration is achieved by the addition of a compound that will directly bond to the water molecules of the Crystallisation buffer that surrounds the crystals. This lowers the number of interactions that proteins can establish with water, forcing it to interact with other molecules, thus inducing conformational changes. Chemicals used can include salts, precipitants and alcohols. From a practical point of view, the researcher either replaces the solution surrounding the crystals samples via careful pipetting or dialysis, or physically transfers the crystals into a new solution. This is the most frequent way of undertaking dehydration and it is often done in combination with cryo-protection or Ligand binding. The key parameters in this process are the chemicals used, their concentration increment (if necessary) and the time samples are allowed to equilibrate in the new solution prior to harvesting (Shi et al. 2008; Adachi et al. 2009).

### Relative Humidity and Its Relationship with Cryo-Protection

Key in dehydration experiments is the concept of Relative Humidity (RH). Relative Humidity is defined as the relative amount of water vapour in a given volume of air. It is expressed as a percentage of the saturation humidity and is pressure- and temperature-dependent (Winston and Bates 1960). As the chemical composition of a solution alters the saturation vapour pressure, it can be used to cross calibrate both the vapour and chemical dehydration experiments. For example at 20 °C a saturated solution of LiCl is tabulated to be around 11 % RH, NaCl 75 % RH, KCl 86 % RH, and K_2_SO_4_ 97 % RH (see Table 6.1 for a more comprehensive list). It is important to note that any standard calibration table applies to closed systems, therefore in devices like the HC1 and FMS that operate using an open airflow, the empirical equilibrium RH values are greater than those presented on Table 6.1 (see also Tables 6.2 and 6.3). For this reason the European Synchrotron Radiation Facility/the European Molecular Biology Laboratory – Grenoble Outstation (ESRF/EMBL) created a web tool ( www.embl.fr/CrystalDehydrationCollaboration/RH.html ) that can be used to estimate the RH of solutions that includes theoretical and empirical values for better reference (Bowler et al. 2015; Wheeler et al. 2012). It is also important to remember that an individual Crystallisation droplet will be in equilibrium with the well solution, but during the course of the crystallisation experiment the well might have dried up over time, thus it is much better to measure the empirical value of each tray before commencing an experiment.

**Table 6.1:** Examples of equilibrium RH for a number of saturated salt solutions (Greenspan 1977)

Saturated salt	RH at 20 °C
Potassium hydroxide	9.32
Lithium chloride	11.31
Potassium acetate	23.11
Magnesium chloride	33.07
Sodium iodide	39.65
Potassium carbonate	43.16
Magnesium nitrate	54.38
Sodium bromide	59.14
Potassium iodide	69.90
Sodium chloride	75.47
Ammonium chloride	79.23
Potassium bromide	81.67
Ammonium sulfate	81.34
Potassium chloride	85.11
Potassium nitrate	94.62
Potassium sulfate	97.59

**Table 6.2:** Examples of empirical equilibrium RH determined for the HC1 for a number non saturated salt solutions from (Bowler et al. 2015)

Concentration (M)	Sodium chloride	Sodium acetate	Sodium malonate	Ammonium sulfate
0.5	99.8	99.9	99.9	99.9
1.0	98.9	98.9	99.3	99.1
1.5	97.2	97.1	97.0	97.7
2.0	95.3	94.8	95.2	95.9
2.5	94.2	93.2	92.5	94.3
3.0	92.8	91.2	89.9	92.8

**Table 6.3:** Examples of empirical equilibrium RH determined for the HC1for a number standard precipitants from (Bowler et al. 2015)

% (w/w)	PEG10000	PEG6000	PEG4000	PEG1500	PEG400	PEG200	Glycerol	Ethylene glycol
10	99.9	99.9	99.9	99.9	99.9	99.9	99.5	98.5
20	99.9	99.9	99.9	99.9	99.9	99.5	96.5	94.0
30	99.9	99.9	99.9	99.9	98.8	97.3	92.0	89.5
40	99.3	99.9	99.2	98.3	96.3	94.9	88.5	85.0

In general, macromolecules tend to crystallise in highly hydrated solutions that require high RH to be stable but lack “cryo-protective” properties. Cryo solutions prevent ice formation by hydrogen bonding to the surrounding water molecules. These bonds also reduce the available water surrounding the crystal structure leading to a desiccation process. The empirical test of several precipitants commonly used in protein Crystallisation clearly shows a correlation between the ability to cryo-protect and lower Relative Humidity (Wheeler et al. 2012). This highlights the intimate link between both effects (dehydration and cryo-protection) on crystal diffraction quality, however, it is difficult to understand the contribution from each of them when assessing diffraction quality collected at cryo-temperatures. Despite the key role dehydration may play in structure determination, researchers often do not carry out systematic studies on the effect it could have on the data quality, thus overlooking the role it might have played in their success or failure.

### Crystal Changes Induced by Dehydration

The beneficial effects of dehydration are firstly, dependent on the original imperfections of crystal structure and then on the possibility of altering them by removing water molecules.

**Fig. 6.1:**
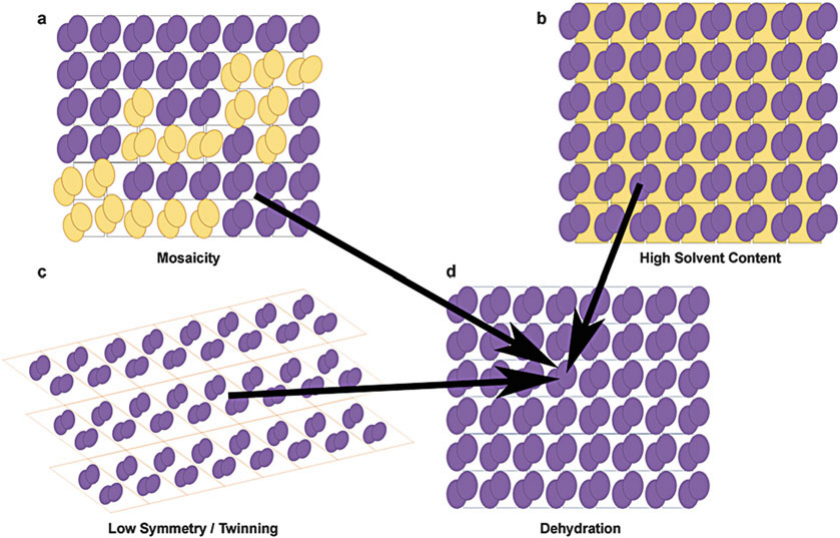
Representation of possible lattice changes induced by dehydration. Most of these changes would lead to increased order or improved protein/solvent ration leading to improved diffraction properties. (**a**–**d**) Represents the reduction of crystal mosaicity. (**b**–**d**)Represents an increased or altered symmetry or the case of reduced twinning. (**c**–**d**) Shows the effect of reduced solvent bulk content leading to improved order or increased protein/solvent ratio

Macromolecular crystals are far from being “perfect”, always presenting a certain degree of mosaicity (meaning they lack of internal periodicity in certain areas called mosaic blocks), see Fig. 6.1. High Mosaic spread in crystals it is mainly caused by the cryo-cooling process and a major contributor to the diffraction intensity reduction. Dehydration can reorganise these mosaic blocks, re-align them, and in principle leading to a crystal with better diffraction properties (Fig. 6.1a).

In other crystals, high solvent content also makes them scatter with less power. If by dehydration, one can lower their global solvent content maintaining the protein molecules order (Fig. 6.1b) (higher protein to solvent ratio) could lead to an improved diffracting power. Changes in solvent content need to be accompanied by small local rearrangements in the crystal contacts and/or flexible areas, therefore diffraction can improve as local motions are reduced and crystal structure changes. In these cases, the degree, direction and the energetics of these changes will condition whether changes are for the better. The actual physical change in the lattice may be subtle, but due to the diffraction phenomenon the results can be very dramatic.

The most dramatic cases are those where the alterations cause enormous difference in the lattice packing triggering a space group change. Often, as the molecules turn and twist, screw axes are generated or removed and alternated indexing solutions start emerging. These alterations might be positive if they induce greater symmetry.

Finally, as it was mentioned early in this section, poor diffraction often is associated with the physical alterations of the crystals when cooled to 100 K. Nevertheless, the cryo-cooling process is very beneficial as it prevents Radiation damage, lowers thermal motions of the molecules, reduces background and in certain cases also improves diffraction resolution. Dehydration prior to cryo-cooling in many cases helps to overcome the issues mentioned above as the pre-contraction induced by the dehydration process can yield a better lattice re-arrangement for the cryo-cooling. In other cases, dehydration is also beneficial as it can act as a way of cryo-protection (Pellegrini et al. 2011). Either by dry mounting, preventing ice forming around the crystals, or by concentrating the solutes within the solvent channels thus preventing hexagonal ice from nucleating in the solvent channels.

### Effect of Dehydration on the Membrane-Protein Crystal Lattice

Membrane proteins can crystallise in two main forms depending on how they are grown. Crystals grown by the Lipid Cubic Phase (LCP) method (Caffrey 2003) tend to be formed by proteins organised in planar layers through protein–Detergents–lipid hydrophobic interactions stacked on top of each other by polar interactions. These crystals are extremely small and fragile two dimensional plates known as type I 3D crystals. These crystals are difficult to harvest, difficult to see and most of their properties are intimately linked to the biochemical properties of the LCP. As the LCP structure is temperature and humidity dependent, this type of crystal is very unlikely to be useful for controlled dehydrations studies. On the other hand, membrane protein crystals can also grow using more “standard” Crystallisation methods such vapour diffusion leading to crystals organised in a more three dimensional fashion (type II 3D crystals) similar to soluble proteins and more suited for dehydration experiments. Despite their similarity with soluble crystals, these membrane protein crystals tend to have lower protein content (10–25 % rather than 45–60 %) and higher solvent content (mainly water in soluble proteins) made up of Detergents/lipids free micelles.

Integrity and dynamics of membrane proteins are closely related to the properties of the surrounding phospholipids in the native membrane. To avoid protein aggregation once removed from its natural environment, Detergents is used in the protein solution in order to mimic the lipid membrane by surrounding the hydrophobic region of the protein generating a water-soluble protein–Detergents complex (PDC). Therefore, membrane protein crystals formation and stability are strongly linked to the protein overall molecular structure (hydrophilic and hydrophobic exposed regions) and to the size, concentration and critical micelle Critical micelle concentration (CMC) (Critical micelle concentration (CMC)) of the Detergents. While some membrane protein crystals are able to tolerate an increase or decrease of Detergents once they have been formed others are more sensitive and lose their order if the equilibrium (bound Detergents/free Detergents) is changed. This will have a strong impact on the crystal dehydration ability. More information in the use of Detergents in membrane protein structure determination can be found in Chap. 10.1007/978-3-319-35072-1_2 of this book.

In soluble protein crystals, dehydration tends to disturb the whole solvent volume and thus induce variation across the entire structure. Here, dehydration can affect solvent exposed areas, flexible loops, crystal contacts and internal hydrogen bonding networks. The idea behind dehydrating these crystals is to induce structural changes that will modify the packing (normally by altering crystal contacts or stabilisation of flexible areas) leading to better diffraction without negatively affecting areas that are already well ordered.

In membrane proteins, most of the interactions that keep the global lattice stable (via the Detergents, lipid or interatomic interactions) are partially fluid and malleable. These regions are mainly hydrophobic and thus not severely affected by dehydration. The few hydrophilic interactions existent act as small anchors that stabilise the lattice. Humidity will affect these small soluble portions and induce changes that can propagate to the whole crystal via rearrangements on the global lattice structure, hopefully inducing a new ordered lattice that will promote better diffraction without affecting the integrity of the protein structure in study.

The magnitude of dehydration to induce a notable change on a crystal structure is, broadly speaking, different between membrane and soluble proteins. In soluble protein crystals, a small change in the global solvent content will cause a huge change in the whole crystal structure therefore these crystals usually require less dehydration. In membrane protein crystals, most of the alterations are targeted at the small solvent rich areas scattered around the lattice, and in order to remove enough water to successfully induce a change on the whole structure, the extent of dehydration needed is often much greater than for soluble proteins. The less amount of water in the lattice, the stronger the process needs to be to extract it. A good example of this are the crystals of the bile acid sodium symporter ASBT (Hu et al. 2011) that have required dehydration from an initial RH of 95 % down to 45 % in order to improve diffraction limit from around 8 Å at room temperature to around 2 Å after cryo-cooling.

Dehydration undertaken on membrane protein crystals without mother liquid in its surroundings (dry mounted samples – see Sect. 6.3.2) only induces changes in water content within the solvent channels. Note that although these channels have great amounts of Detergents and other solutes there is very little water available for the dehydration process. On the contrary, dehydration methods that are performed on wet samples surrounded by their mother liquor (see Sect. 6.3.1) affect the whole drop. As water content is higher, dehydration will induce an increased concentration of solutes and Detergents surrounding the crystals that can in turn alter the equilibrium with the crystal and disturb its order. For these reasons it is worth trying both wet-mounting and dry-mounting dehydration methods.

## Tools and Techniques for Crystal Dehydration

Practical considerations and timing, determine what most researchers can do with their samples. Crystal availability and beam time do not always match and researchers will always struggle to get their best crystals ready in time for a dehydration study at a synchrotron or home source. In this section, the available dehydration methods and tools have been separated by the ability to execute them in a standard laboratory or at an X-ray source.

### Dehydration in the Laboratory

Doing dehydration at the home laboratory has its advantages. The process can be performed using standard tools available within any molecular biology or biochemistry laboratory and with total control of the researcher. This also allows the researcher to test a great number of crystals (if available) without the usual time constrains during a synchrotron visit. In general, when dehydrating crystal samples in the home laboratory, the dehydration procedure is the most basic one where samples are just transferred from one solution to the other or exposed to air for a few seconds before proceeding to the cryo-protection process. This way of dehydrating is normally undertaken casually, not regarded important and often not well described in the methods sections of publications.

**Fig. 6.2:**
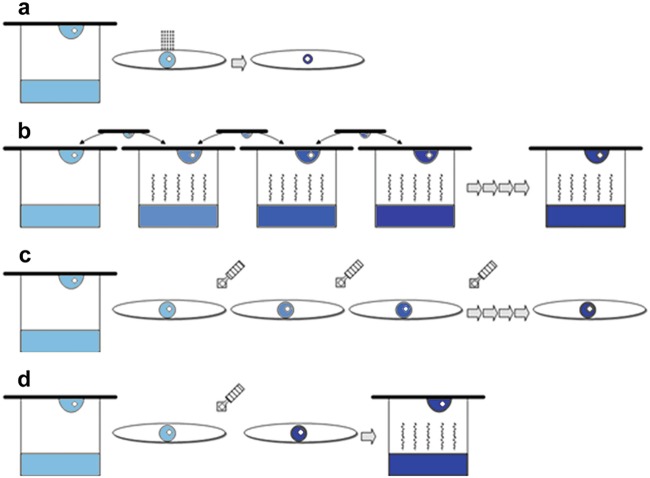
Diagram showing alternate options for laboratory dehydration. (**a**) Simple air exposure, (**b**) vapour control by exchanging well solution, (**c**) chemical exchange by serial soaking and (**d**) chemical exchange by soaking and slow equilibration

The most common dehydration techniques used in a standard laboratory are described below, see also Fig. 6.2.

#### Simple Air Exposure

This is the simplest dehydration method consisting in opening the Crystallisation plate allowing the drops to dry for a certain amount of time prior to cryo-cooling (Fig. 6.2a). It is, of course, dependent on the ambient RH, temperature, size of the drops and chemical composition of the crystallisation solution. It is mainly responsible for the “I am getting better at harvesting crystals” syndrome, as with time, crystallographers either start harvesting faster (allowing for less dehydration in delicate samples) or they harvest numerous samples from an individual drop (allowing for the last ones to dehydrate more). It can also be induced during the final harvesting prior cryo-cooling by allowing the sample to dehydrate on the loop before the final plunge in liquid nitrogen. Uncontrolled dehydration is difficult to avoid but careful note taking and repetition is the key to getting to control or understand its effects. As crystals become more fragile, smaller and grown in smaller volumes the effect of dehydration by air exposure becomes more prevalent.

#### Vapour Control by Exchanging Well Solution

This is probably the most controllable way of undertaking dehydration in the laboratory. It is, for obvious reasons, only possible in vapour diffusion Crystallisation samples and relies on using the same crystallisation drop for the actual experiment. The idea is to replace the well solution with increased amounts of a desiccating solution (Fig. 6.2b). The new solution will lower the water vapour pressure and in return “pull” water out of the crystallisation drop inducing a change in the crystals. This is normally undertaken by using some of the standard saturated calibration salt solutions but can also be done using crystallisation precipitants. Depending on the volumes available, dehydration can be achieved by simple addition of salts to the pre-existing well buffer or by replacing the well with a totally new solution. Dehydration is a slow and gradual process most of the time, which depends on the drop size and well volume. It can be undertaken in one single step or in progressive steps and normally, after a period of equilibration (normally between 8 and 24 h), samples are harvested and stored for data collection. The most attractive feature of this dehydration method is its simplicity and easy implementation. It can be undertaken whenever samples are available and once the optimal conditions are found it is very easy to reproduce. It can be achieved at any temperature and in general the presence of highly volatile compounds in the Crystallisation solutions is not a concern.

#### Dehydration by Chemical Exchange of Crystallisation Droplet

As discussed earlier, this process is potentially happening during the standard cryo-cooling or soaking procedures (Fig. 6.2c,d). The main difference between this and the previous method described (Sect. 6.3.1.2) is that here it is possible to change the chemical components of the crystallisation drop and not only the concentration of the pre-existing ones. This procedure opens the possibility that the chemical used for dehydration alters the structure and/or the resulting diffraction by chemical modification rather than dehydration. In addition, the rate of exchange is more difficult to control and normally carried out by slow diffusion of buffers with increased concentration. If Crystallisation droplets are very small, it is easy to cause a shock on the crystals, therefore pre-mixing solutions to the target concentration can sometimes help. The simplest way of chemical dehydrating is by dialysis. This only requires replacing the solutions and, due to the dialysis membrane, it is very gentle and controllable.

### Modern Dehydration Methods with X-ray Feedback

#### Capillary Dehydration

Dehydration performed in home laboratories, despite being the simple and cost effective, it is a partially blind process by the fact that is decoupled from the X-ray data collection. In addition, the cryo-cooling process undertaken after the dehydration procedure can muddle results.

**Fig. 6.3:**
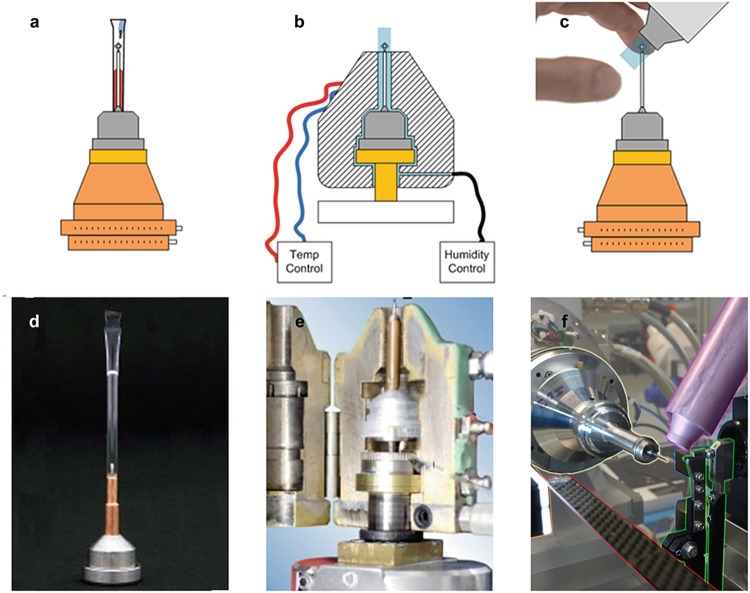
Methods of dehydration with X-ray feedback. (**a**) and (**d**) Capillary dehydration. (**b**) and (**e**) Free Mounting System. (**c**) and (**f**) The humidity control dehydration device (HC1)

This was noted early on in the use of macromolecular crystallography and initial attempts were made by developing tools that would couple salt-based vapour dehydration to the, then available, data collection using the capillary (glass or quartz) mounting method (Huxley and Kendrew 1953; Pickford et al.^1993^; Kiefersauer et al. 2000a, b). For a time, this proved to be useful (Rockland 1960; Dobrianov et al. 2001) but fell into disuse due to the high X-ray scatter background produced compared to loops. The technique was re-introduced a few years later (Mac Sweeney and D’Arcy 2003; Basavappa et al. 2003) by adapting new lower background capillaries (made of a transparent polymer) to the then standard pin-based mounts (Kalinin and Thorne 2005; Kalinin et al. 2005; Hellert et al. 2014) (Fig. 6.3a,d). The later method is cheap, easy to implement and very popular amongst researchers attempting to dehydrate whilst collecting X-ray data *in-house* and for simple cryo-cooling (Yao et al. 2004; Kim et al. 2007). As the mounts (MicroRT system – MiTeGen) are standard, the technique is also available at any synchrotron beamline where manual mounting is possible. It is most frequently used as a quick dehydration method and as an easy way of keeping samples stable during room temperature data collections.

The process involves pre-loading (using a gel-loading tip) a transparent polymer capillary that is sealed at one end with a small volume of stabilising or dehydrating solution. Crystals are harvested with a loop or mesh mounted onto a pin and then covered with the capillary through the open end such that it seals. The sample is maintained at a close distance from the liquid. The pin can then be mounted and data collected through the capillary whilst the crystal is kept stable or whilst being dehydrated. The dehydration rate is dependent on the solution composition, the sample volume, the solution volume and the distance between the sample and solution. Cryo-cooling is possible at any point by quickly removing the capillary and plunging in liquid nitrogen (Kalinin and Thorne 2005; Warkentin et al. 2008).

#### Dehydration Using the FMS

The Free Mounting System (FMS) was the first device able to automate the process of dehydration. It operates based on the alteration of RH by the adjustment of the dew point and is able to regulate humidity in a crystal from 98 % to 50 % at temperatures ranging from 15 °C to 25 °C. It has been used in some academic laboratories and private companies yielding interesting results (Kiefersauer et al. 2000a, b).

The key feature of the device is a holding block (Fig. 6.3b,e) that is able to keep the temperature of the sample and the surrounding airflow stable. The samples are mounted onto meshes or loops and placed inside the block. The block is then placed over a standard goniometer mount to allow users to centre the samples onto the X-ray beam path and collect data whilst dehydrating samples.

The major advantage of this device over classic methods is that complex protocols such gradient dehydration, rehydration and reverse hydration can be achieved with a high level of reproducibility while at the same time allowing diffraction feedback. The only disadvantage compared to the classic method is the fact that only one or two crystals can be analysed at the same time.

The FMS has been used in a number of successful cases, most notably the improvement in diffraction of the F1-ATPase protein (Bowler et al. 2006). It also has allowed for the determination of the extremely interesting case of carbon monoxide oxidase (CODH) (Kiefersauer et al. 2000a, b) that required a complex dehydration-rehydration process only possible with the use of the FMS.

Despite its success, the complexity of sample handling and bulky design have prevented FMS from being widely used in laboratories worldwide and also making synchrotrons sources shy away from installing it on macromolecular crystallography (MX) beamlines. Lately the developers of the device have also proved it can be used in combination with a heating Lasers. This gives rise to new alternate options to automate dehydration and new cryo-cooling methods, but no new crystal structures have as yet resulted from this sort of work (Kiefersauer et al. 2014).

#### Dehydration Using the HC1 System

The humidity control dehydration device (HC1) was developed a few years after the FMS in order to address the factors limiting the wider use of this method by the community (Sanchez-Weatherby et al. 2009; Russi et al. 2011). The HC1 key difference is that, despite also being based on dew point, the delivery of humid airflow is achieved via a Nozzle rather than a cooling block (Fig. 6.3c,f). The HC1 Nozzle makes it unable to alter the temperature of the air around the sample but makes it very easy to install and use it in a standard academic laboratory with a home X-ray source or any MX synchrotron beamline. The low complexity of the device reduced the cost and simplified its maintenance making it better received by many academic laboratories and a large number of synchrotron beamlines around the world. To date nine devices are available at several world synchrotrons. The ESRF/EMBL/ILL operate two devices available across the MX beamlines. The Helmholtz-Zentrum Berlin (Mueller et al. 2012) and Max IV Laboratory (Ursby et al. 2013) operate the device installed in one of their MX branches. In the US, both APS (NE-CAT 24-ID-E) and the ALS (Berkeley Center for Structural Biology beamlines) have one each, the Canadian Light Source (Fodje et al. 2014) has one and Diamond Light Source in the UK has another two that can be both installed in any of the MX beamlines and also in a laboratory as an off-line setup.

Considerable efforts have been made at several of the synchrotrons in order to incorporate the HC1 within their hardware and software. For example, workflows within MxCuBE (Gabadinho et al. 2010) have been adapted to undertake dehydration experiments (Brockhauser et al. 2012) and the Diamond beamline software (GDA) now includes a bespoke data collection perspective solely dedicated at undertaking these sorts of experiments. This allows synchrotron users to be able to undertake a range of dehydration experiments as part of their normal beamtime allocation. In some synchrotron facilities (BM14 at the ESRF and I02, I03 and I04 at Diamond), installations also include rapid swapping between the use of the HC1 and the standard cryo-temperature allowing constant routine use without the local beamline support. Others (like MaxLab and BESSY) have opted for a more permanent installation (Mueller et al. 2012; Ursby et al. 2013).

Either in the laboratory or at the synchrotron beamline, any HC1 experiment requires the mounting of samples in the airflow at their starting Relative Humidity (RHi). The relative humidity is empirically determined by observing the behaviour of a droplet solution (Crystallisation solution) under the humid airstream while varying the RH. Once the optimum humidity has been determined, crystal samples can be mounted using micromeshes (MiTeGen) so that any excess buffer can be removed by wicking. This makes the experiments more reproducible and ensures samples don’t move during the process.

Dehydration protocols can then be applied to each sample by starting at the RHi and lowering humidity to desired target points. At these points samples can either be directly analysed with X-rays or cryo-cooled for later analysis. The key to any experiment is to try to induce changes in the crystal lattice that will yield a better diffracting sample. Once a potential dehydration protocol has been determined then a number of crystals can be dehydrated and cryo-protected to find the elusive structure. If available, this final harvest can be undertaken in a laboratory in order to save precious beam time and improve throughput.

When using the HC1 in a beamline, as it is a room temperature experiment, it is important to try to separate the effects from dehydration from the expected effects of radiation damage. A burn test may be appropriate to define a baseline. The best way of following an on-line dehydration experiment is to collect weak, wide oscillation images (1–2°) that will allow manual indexing to determine lattice dimensions and mosaicity. Following the changes in the lattice, the researcher can determine any transition points of interest that could suggest points of improved diffraction.

If undertaking the experiment in the laboratory it is important to also harvest some control samples under known conditions and try to ensure samples have had enough time to undergo any transition (10–15 min at least) prior to cooling. Harvesting a number of repeats helps to assess the true final outcomes of the process giving an indication of the likeliness of the method in study.

#### *In situ* Dehydration

**Fig. 6.4:**
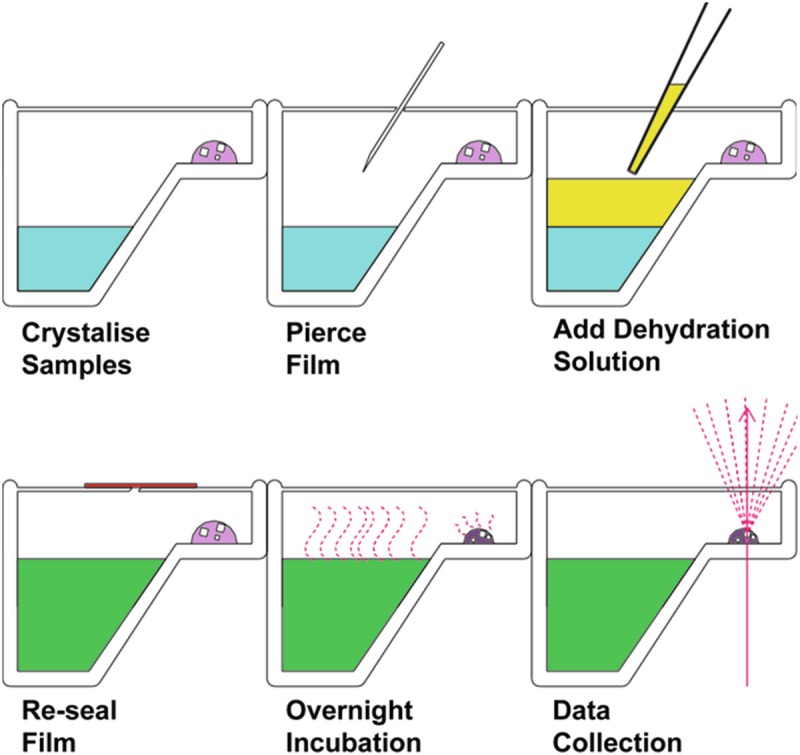
Diagram showing the different stages required for an *in situ* dehydration experiment

In most of the MX beamlines across the world, sample environments (also called experimental hutches) have been made more automation-friendly. Consequently, the space available for additional and special setups became more restrictive. Despite this, due to the developments of photon counting pixel array detectors and their potential to help outrun radiation damage (Kraft et al. 2009; Aishima et al. 2010; Rajendran et al. 2011) there have been a number of technical upgrades to allow data collection of room temperature samples within these crowded spaces. These include new Crystallisation plates (with lower profile and better geometry), upgraded goniometry, dedicated robotics and imaging systems, make room temperature data collection possible. The option of collecting data directly from crystallisation plates without the need of any physical manipulation of the crystals brings new links between the experiment in the laboratory and the X-ray source. Essentially, in this method, crystals from the same crystallisation condition are grown in a full 96-well crystallisation plate (always advisable to use Crystallisation plates with flat bottoms to avoid visual artefacts when mounted at the beamline). Once crystals are grown, gradients of different dehydration solutions are added to the plate wells through small pierces (without touching the crystal drops) and re-sealed (see Fig. 6.4). The dehydration is allowed to proceed for a period of 8–12 h before the plate be taken to an X-ray source with capabilities for In situ plate screening (Douangamath et al. 2013; Moraes and Archer 2015; Axford et al. 2015). This method is essentially a vapour control, by exchanging well solutions, which allows multiple samples to be dehydrated in a number of ways in one single procedure. With high throughput tools it is possible, with a reasonable amount of beam time, to analyse these complex dehydration results without the blurring effect of cryo-cooling. In summary, the fundamental advantages of using *in situ* is that there is no need for direct crystal handling and therefore small or delicate samples are not a problem, crystals can grow at 4 °C with the procedure carrying on at the same temperature and there is no need for cryo-cooling.

In these sorts of experiments, the key is to analyse large populations of crystals and observe general trends of change amongst them. These experiments are analysed by studying changes in lattice dimensions, mosaicity, resolution and diffraction properties as a function of the chemical composition of wells. The nature of the initial cohort of crystals can be well defined; therefore their susceptibility to change, any transition points, any regions of stability and a minimum lattice size should all be possible using limited beamtime. Once points of interest are identified, a sub-population of test samples can then be harvested and cryo-cooled in order to confirm any beneficial effects. These cryo-cooling tests should help define a best protocol for dehydration and cryo-protection.

#### Analysing Results and Making Decisions

As it has been described throughout this chapter, there are a number of valid options when carrying out dehydration. The researcher’s choice will depend on a number of constraints such sample, resources and time availability (see Table 6.4). Also depending on the method used the outcomes will vary. In certain occasions, observations will be immeasurable due to crystal cracking, shrinking or dissolving, in others observations will be easy to measure (lattice dimensions, mosaicity and resolution limits) and unfortunately, in others negative results will be the outcome (lack of diffraction, presence of ice rings or non indexable lattices). For these reasons it is advisable to correlate all the different information available and try to make sense of partial data. All the different types of dehydration experiments can be linked via their relation to Relative Humidity. By using available tabulated information (salt dehydration) or measuring empirical humidity (HC1/FMS) researchers can correlate results from different experiments. The idea is to try to extract as much information and try new experiments that may lead to a positive outcome with the available tools. For example, prior to any dehydration attempt, most researchers have already tried standard cryo-cooling, soaks and handled their crystals in a number of ways. Researchers are familiar with crystals stability under the microscope and their methods for soaking in cryo-protectant are useful when planning dehydration protocols either using the HC1/FMS or a vapour diffusion experiment. Similarly, measuring or calculating the equilibrium RH of these solutions can give to the crystallographer clues of why some of the solutions are better than others and suggest new cooling protocols. Often, cryo diffraction data is also available and so a quick comparison with room temperature data collection can possibly yield fruitful clues. Variable indexing in cryo lattices can be an indication of a varied initial crystal population suggesting a variable response to cryo making crystals interesting candidates for dehydration. A review of old crystal forms observed during past experiments (despite not diffracting well) can also give useful clues to plan dehydration experiments. During dehydration, the observation of lattice transitions, minimum lattices and points of loss of diffraction can be used later when defining starting points for cryo-cooling methods. Even dehydration itself can be used as a simple cryo-protecting strategy useful in preventing adverse effects of penetrative cryo-protectant (Pellegrini et al. 2011). Using Relative Humidity as a way of cross-referencing results from the different experiments allows decisions to be made on quantitative measurements and permits experiment planning that will yield easier to compare results.

**Table 6.4:** Comparative of the different properties of the possible dehydration methods

Dehydration method	Reproducible	Gentle dehydration	Fast dehydration	Temperature control	Automated	Reversible	Volatiles	High throughput	Direct X-ray feedback	No handling required	Low cost/ accessible
Simple air exposure	×	×	✓	✓	×	×	×	×	×	×	✓
Vapour control by exchanging well solution	✓	✓	×	✓	×	✓	✓	✓	×	×	✓
Chemical exchange of Crystallisation droplet	×	✓	✓	✓	×	✓	✓	✓	×	×	✓
Capillary dehydration	✓	✓	×	×	×	✓	✓	×	✓	×	✓
Free mounting system	✓	✓	✓	✓	✓	✓	×	×	✓	×	×
HC1 dehydration	✓	✓	✓	×	✓	✓	×	×	✓	×	×
In situ dehydration	✓	✓	×	✓	×	✓	✓	✓	✓	✓	✓

As it was mentioned earlier, when attempting dehydrating membrane protein crystals it is worth keeping in mind the particular packing of these crystals and how this might affect the results. Detergents micelle size/concentration and limited hydrophilic contacts between the molecules in the crystal lattice should be the main points to take into account during interpretation of results. In addition, as these crystals have larger solvent channels, often require greater dehydration than required for soluble proteins. Therefore, one should include both vapour diffusion and chemical modification methods as they might yield markedly different results. Finally, given the fragile nature these crystals, they are likely to benefit from handling free techniques such as in situ dehydration and dialysis.

## Conclusions: Success in Dehydration

Unfortunately due to a multitude of constraints in most research projects, controlled dehydration is often not attempted or just coarsely tested. Researchers frequently associate the positive outcomes of dehydration with the time required for the experiment. Later, by asking colleagues and consulting the available literature often conclude that it is unlikely to be useful for them. This makes dehydration very much a last resort technique, in particular when more standard approaches have failed and the project is important enough to pursue alternatives. In addition, it is known that many of dehydration success cases are not reported in methods sections of scientific papers and it is considered of less importance.

With regard to membrane proteins, a recent review (Bowler et al. 2015; Russo et al. 2012) on dehydration showed that despite the large number of soluble protein structures solved compared with membrane proteins, the number of reported membrane protein targets facilitated by dehydration seem to be very similar to soluble proteins, suggesting that success is inclined towards membrane proteins. It is difficult to be sure but three potential explanations for this phenomenon are:

- Membrane proteins are more costly (in time and money) to produce and investigators may be willing to try every available technique possible before moving onto other projects
- The nature of the membrane protein crystals themselves could be the blame. Membrane protein crystals have smaller water content but overall higher solvent content facilitating motions across the solvent channels making them more susceptible to improvement by dehydration
- It seems that a membrane protein structure which is facilitated by a dehydration method always has a reference within the results section of the scientific paper as it is perceived to be important for the research field

Whatever the reason, it is clear that undertaking controlled dehydration in membrane protein crystals is well worth the effort in most cases.
